# Interest in “organic,” “natural,” and “additive-free” cigarettes after hearing about toxic chemicals in cigarette smoke

**DOI:** 10.1371/journal.pone.0212480

**Published:** 2019-03-06

**Authors:** Sabeeh A. Baig, M. Justin Byron, Jessica K. Pepper, Noel T. Brewer

**Affiliations:** 1 Department of Sociomedical Sciences, Columbia University Mailman School of Public Health, New York, New York, United States of America; 2 Department of Health Behavior, UNC Gillings School of Global Public Health, Chapel Hill, North Carolina, United States of America; 3 Department of Family Medicine, UNC School of Medicine, Chapel Hill, North Carolina, United States of America; 4 Center for Health Policy Science & Tobacco Research, RTI International, Research Triangle Park, North Carolina, United States of America; Brown University, UNITED STATES

## Abstract

**Introduction:**

The US Family Smoking Prevention and Tobacco Control Act requires the government to disseminate information about the toxic chemicals in cigarette smoke. We sought to understand how the descriptors “organic,” “natural,” or “additive-free” affect smokers’ interest in cigarettes in the context of information about chemicals in cigarette smoke.

**Methods:**

Participants were a national probability sample of 1,101 US adult (ages ≥18) smokers recruited in 2014–2015. A between-subjects experiment randomized participants in a telephone survey to 1 of 4 cigarette descriptors: “organic,” “natural,” “additive-free,” or “ultra-light” (control). The outcome was expected interest in cigarettes with the experimentally assigned descriptor, after learning that 2 chemicals (hydrogen cyanide and lead) are in cigarette smoke. Experimental data analysis was conducted in 2016–2017.

**Results:**

Smokers indicated greater expected interest in “organic,” “natural,” and “additive-free” cigarettes than “ultra-light” cigarettes (all *p* <.001) after learning that hydrogen cyanide and lead were in cigarette smoke. Smokers who intended to quit in the next 6 months expressed greater expected interest in the 4 types of cigarettes (“organic,” “natural,” “additive-free,” and “ultra-light”) compared to smokers not intending to quit (*p* <.001).

**Conclusions:**

Smokers, especially those intending to quit, may be more inclined towards cigarettes described as “organic,” “natural,” and “additive-free” in the context of chemical information. An accumulating body of evidence shows that the US should fully restrict use of “organic” and “natural” descriptors for tobacco products as it has done for “additive-free” and “light” descriptors.

## Introduction

The 2009 Family Smoking Prevention and Tobacco Control Act requires the US to inform the public about the toxic chemicals in cigarette smoke [1]. Messages that communicate this information could have the unintended consequence of steering smokers and susceptible non-smokers towards cigarettes described as “organic,” “natural,” or “additive-free.” Some consumers have the misperception that these cigarettes are less harmful [2–4], even though no cigarettes are safer than any others [5]. Before the terms were banned in 2010 [6], consumers were similarly misled by cigarettes marketed as “low-tar” and “light” [7]. Concern about cigarettes marketed as “natural” is important because the brand Natural American Spirit has rapidly grown in market share in recent years [8]. While some tobacco companies recently agreed to cease using the descriptor “additive-free,” the US still allows the descriptor “organic,” and the descriptor “natural” in the “Natural American Spirit” brand name) [9]. We sought to understand how the terms “organic,” “natural,” or “additive-free” affects smokers’ interest in cigarettes, in the context of information about the chemicals in cigarette smoke.

## Methods

### Participants, procedures, and measures

The Carolina Survey Research Laboratory recruited a national probability sample of 5,014 US adults (ages ≥18) using random digit dial landline and cell phone frames from September 2014 through June 2015. Additional details on survey methods are available elsewhere [10]. Interviewers obtained verbal consent from participants. The Institutional Review Board at the University of North Carolina approved the study.

Current smokers were participants who had ever smoked at least 100 cigarettes and now smoke every day or some days [11]. Of the 1,151 smokers who completed the survey, we analyzed data from 1,101 with complete data. Current smokers were randomized to one of 4 cigarette descriptors: “organic,” “natural,” “additive-free,” or “ultra-light.” We chose “ultra-light” as the control because this descriptor is well established as being misleading [7] and, unlike the term “light” [12] few smokers would identify their current cigarettes using this descriptor. Experimental condition did not differ by participant characteristics (all *p*≥.74), confirming randomization was successful.

The survey assessed self-reported increase in interest (expected interest) in cigarettes with the experimentally assigned descriptor: “If you learned that chemicals like lead and hydrogen cyanide are in cigarette smoke, how much would that increase your interest in [organic/natural/additive-free/ultra-light] cigarettes?” Response options were “not at all” (coded as 0), “a little” (1), “somewhat” (2), and “a lot” (3). We cognitively tested this survey item and piloted it on Amazon Mechanical Turk before use in the survey. The survey also assessed demographic characteristics and whether participants currently smoked cigarettes they believed to be “light” or equivalent (i.e., “ultra-light”, “mild,” “gold,” or “silver”) [13], currently smoked some days or every day, and intended to quit smoking within the next 6 months.

### Statistical analysis

We used one-way analysis of variance (ANOVA) to examine the impact of descriptor on the primary outcome and between-subjects post-hoc *t*-tests to compare specific conditions. We used two-way ANOVA to examine currently smoking “light” or equivalent cigarettes, currently smoking every day, and intending to quit within the next 6 months as potential moderators of the impact of cigarette descriptor. Analyses were conducted using R (v. 3.5.1) [14] in 2016–2017, two-tailed tests and a critical alpha of.05 except for post-hoc *t*-tests that used Bonferroni adjustments to critical alpha.

## Results

The majority (72.1%) of participants smoked every day, and 33.4% smoked “light” or equivalent cigarettes (Table 1). More than three-quarters (76.8%, 95% CI: 74.2–79.3) of participants indicated at least “a little” expected interest in cigarettes with the experimentally assigned descriptor after learning that lead and hydrogen cyanide were in cigarette smoke. The majority of smokers also indicated at least “a little” expected interest in each of the 4 descriptors: “ultra-light” cigarettes (62.3%, 95% CI: 56.2–68.1), “organic” cigarettes (77.2%, CI: 72.2–81.7), “natural” cigarettes (86.0%, CI: 81.2–90.0), or “additive-free” cigarettes (82.4%, CI: 77.1–86.9).

**Table 1:** Participant characteristics (unweighted).

	Adult *n* = 1,101%
Age (years)	
18–25	15.2
26–34	18.3
35–44	19.6
45–54	23.5
55–64	16.3
65+	7.1
Female	48.1
Race	
White	69.1
Black	20.4
Native American	3.9
Asian	1.0
Other	5.6
Hispanic	6.5
Gay, lesbian, or bisexual	5.5
Education	
< high school	17.9
High school degree or equivalent	35.1
Some college	22.3
Associate’s degree	10.3
College degree	11.1
Master’s degree	3.3
Smoke every day	72.1
Smoke “light” or equivalent cigarettes	33.4
Intend to quit within the next 6 months	46.4

Note. “Light” or equivalent cigarettes includes smokers of “light,” “ultra-light,” “mild,” “gold,” or “silver” cigarettes.

**Fig 1:**
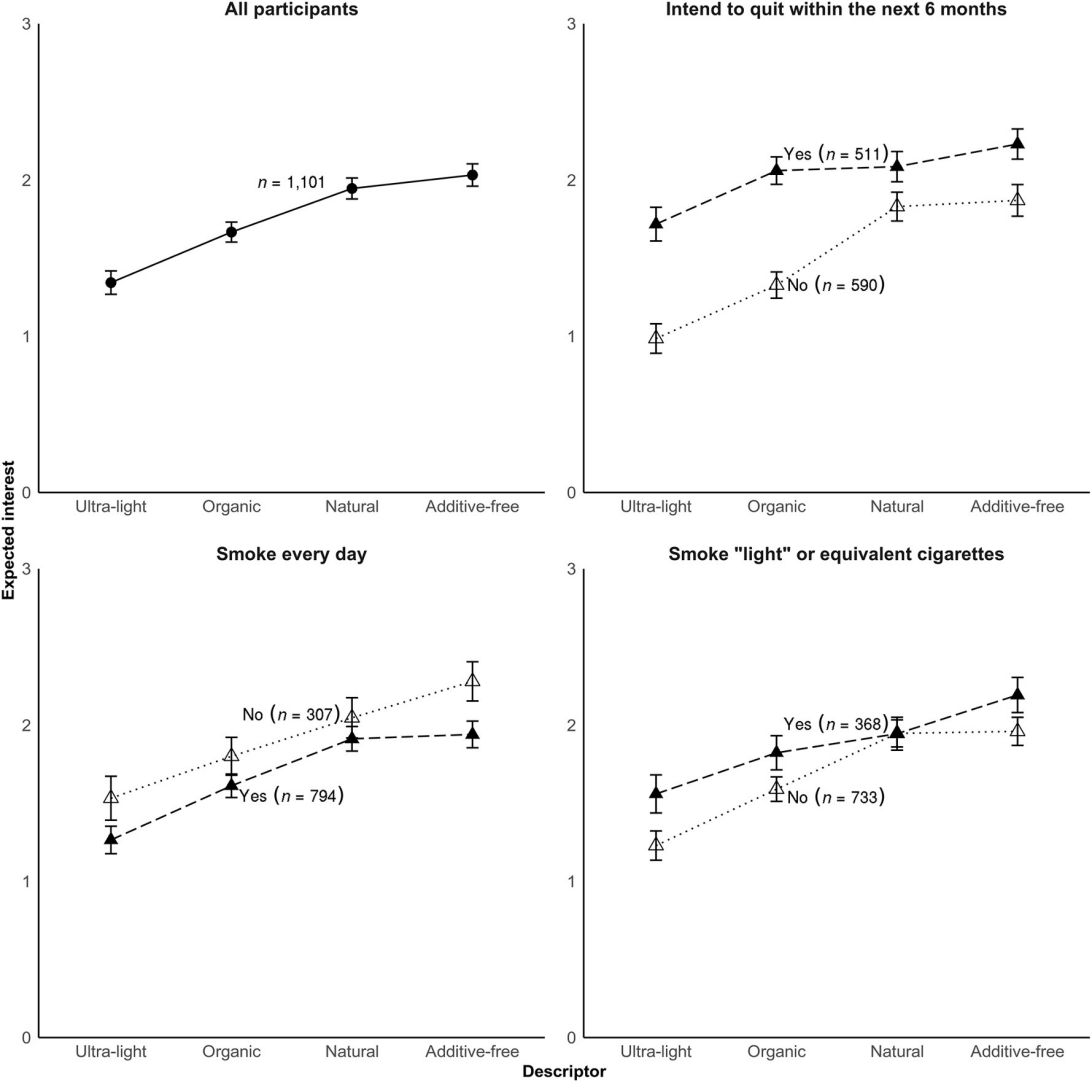
Expected interest in “ultra-light,” “organic,” “natural,” and “additive-free” cigarettes after learning about the toxic chemicals in cigarette smoke. Error bars show simple standard errors.

Expected interest was lowest for “ultra-light” (control) cigarettes (*M* = 1.34, *SD* = 1.22) and higher for “organic” (*M* = 1.67, *SD* = 1.15; *p* = .001). Expected interest was even higher for “natural” (*M* = 1.95, *SD* = 1.08; *p* = .003) which did not differ from “additive-free” (*M* = 2.03, *SD* = 1.13; *p* = .38). Expected interest varied by cigarette descriptor (*F*(3, 1,097) = 19.3, *p* <.001; Fig 1).

Smokers who intended to quit within the next 6 months expressed greater expected interest (*M* = 2.02, *SD* = 1.11) in the 4 cigarette types than those who did not (*M* = 1.49, *SD* = 1.18; *p* <.001). Quit intentions moderated the effect of descriptor (*F*(3, 1,093) = 3.39, *p*_interaction_ = .02; Fig 1). Among smokers who did not intend to quit, descriptors affected expected interest in the same rank order (“ultra-light” < “organic” < “natural” = “additive-free”) as for the full sample (all *p* <.01). Among smokers who intended to quit, the pattern was slightly different, with “organic” also being similar to “natural” (*p* = .85). Frequency of smoking (*p* = .82), smoking “light” or equivalent cigarettes (*p* = .44), and demographic characteristics (all *p*≥.32) did not moderate the impact of descriptor.

## Discussion

The descriptors “organic,” “natural,” and “additive-free” led to higher interest in cigarettes while learning about toxic chemicals in cigarette smoke. This finding was consistent regardless of intent to quit within the next 6 months, frequency of current smoking, and use of “light” or equivalent cigarettes. This finding also did not differ by demographic characteristics. In general, expected interest was lower for “organic” than “natural” or “additive-free” cigarettes. This pattern follows from prior studies showing that some people are skeptical of “organic” descriptors [15], but clearly think that additives are the main source of harm from smoking [2,16,17]. The only exception to the pattern was participants intending to quit within the next 6 months, who did not show a difference in expected interest between “organic” and “natural” or “additive-free” cigarettes, potentially because their quit intentions were motivated by health concerns that overrode any skepticism toward “organic” descriptors.

Descriptors of “organic,” “natural,” and “additive-free” may steer some smokers, especially those intending to quit, toward putatively “healthier” cigarettes rather than encouraging quitting. In a previous experiment, we found that “organic,” “natural,” and “additive-free” descriptors reduced the perceived harm of cigarettes and increased interest in switching to cigarettes with those descriptors [18]. We speculate that, in the current study, these descriptors may have similarly reduced perceptions of cigarettes’ harm, making these cigarettes more appealing in the context of new information about chemicals. Some cigarette promotion appears to capitalize on the public’s misunderstanding about these chemicals.

### Limitations

Strengths of our study include the experimental design and large national probability sample of adult smokers. A limitation is that the experiment relied on a hypothetical scenario, although this is a useful strategy for exploring new research topics. Other limitations include that we did not directly manipulate the presence of chemical information, we did not ask whether the information might decrease their interest, and we did not study adolescents. Future experiments should directly test the unintended consequences of communicating chemical information among adults by manipulating exposure to such information; identify any associated underlying psychological mechanisms; and determine whether information about chemicals has similar effects in at-risk populations like adolescents.

## Conclusions

A growing body of literature shows that “organic,” “natural,” and “additive-free” descriptors are misleading [3,4,15,19–22]. These findings take on new importance in the context of federal law requiring disclosure of information about the toxic chemicals in cigarette smoke. Misleading cigarette descriptors may be especially worrisome without contextualizing information that toxic chemicals are in the smoke from all cigarettes. In the Family Smoking Prevention and Tobacco Control Act, the government restricted “light,” and in the recent settlements, “additive-free” has also been restricted. It is now appropriate to likewise fully restrict the terms “organic” (and “natural” in the brand name Natural American Sprit) for offering smokers a false sense of reduced harm.

## References

[1] 111th Congress of the United States of America. Family smoking prevention and tobacco control act. Public Law 111–31, 123 Statute 1776; 2009.

[2] Morgan JC, Byron MJ, Baig SA, Stepanov I, Brewer NT. How people think about the chemicals in cigarette smoke: A systematic review. J Behav Med. 2017;40(4):553–64. 10.1007/s10865-017-9823-5 28224264 PMC5501992

[3] Leas EC, Ayers JW, Strong DR, Pierce JP. Which cigarettes do Americans think are safer? A population-based analysis with wave 1 of the PATH study. Tob Control. 2016;26(e1):e59–60. 10.1136/tobaccocontrol-2016-053334 27742918 PMC5858197

[4] Pearson JL, Johnson A, Villanti A, Glasser AM, Collins L, Cohn A, et al Misperceptions of harm among natural american spirit smokers: Results from wave 1 of the population assessment of tobacco and health (path) study (2013–2014). Tob Control. 2016;26(e1):e61–7. 10.1136/tobaccocontrol-2016-053265 27924008

[5] US Department of Health and Human Services. The health consequences of smoking–50 years of progress: A report of the surgeon general. Atlanta, GA: US Department of Health; Human Services, Centers for Disease Control; Prevention, National Center for Chronic Disease Prevention; Health Promotion, Office on Smoking; Health; 2014.

[6] Campaign for Tobacco Free Kids. Ban on deceptive cigarette labels “light” and “low-tar” takes effect June 22. 2010 [Internet]. https://www.tobaccofreekids.org/press_releases/post/id_1216

[7] US Department of Health and Human Services. Risks associated with smoking cigarettes with low machine-measured yields of tar and nicotine. Bethesda, MD: US Department of Health; Human Services, Public Health Services, National Institutes of Health; 2001.

[8] Crave R. Reynolds’ super premium cigarette taking off [Internet]. 2015. http://www.journalnow.com/business/business_news/local/reynolds-super-premium-cigarette-taking-off/article_a6cad0d5-c2fd-5cc5-8307-894d15415551.html

[9] Campaign for Tobacco Free Kids. FDA/Santa Fe Natural Tobacco agreement fails to protect the public from misleading claims and imagery on natural American spirit cigarettes [Internet]. 2017. https://www.tobaccofreekids.org/press_releases/post/2017_03_02_fda

[10] Boynton MH, Agans RP, Bowling JM, Brewer NT, Sutfin EL, Goldstein AO, et al Understanding how perceptions of tobacco constituents and the FDA relate to effective and credible tobacco risk messaging: A national phone survey of U.S. adults, 2014–2015. BMC Public Health. 2016;16(1):516 10.1186/s12889-016-3151-5 27333921 PMC4918079

[11] Davis S, Malarcher A, Thorne S, Maurice E, Trosclair A, Mowery P, et al State-specific prevalence and trends in adult cigarette smoking-United States, 1998–2007. MMWR-Morbid Mortal W. 2009;58(9):221–6. 19282813

[12] Cornelius ME, Cummings KM, Fong GT, Hyland A, Driezen P, Chaloupka FJ, et al The prevalence of brand switching among adult smokers in the USA, 2006–2011: Findings from the ITC US surveys. Tob Control. 2014;24(6):609–15. 10.1136/tobaccocontrol-2014-051765 25260750 PMC4743742

[13] Connolly GN, Alpert HR. Has the tobacco industry evaded the FDA’s ban on ‘light’ cigarette descriptors? Tob Control. 2013;23(2):140–5. Available from: 10.1136/tobaccocontrol-2012-050746 23485704 PMC3932763

[14] R Core Team. R language definition. Vienna, Austria: R foundation for statistical computing 2000

[15] Byron MJ, Baig SA, Moracco KE, Brewer NT. Adolescents’ and adults’ perceptions of ‘natural’, ‘organic’ and ‘additive-free’ cigarettes, and the required disclaimers. Tob Control. 2015;25(5):517–20. 10.1136/tobaccocontrol-2015-052560 26628496 PMC4887411

[16] Brewer NT, Morgan JC, Baig SA, Mendel JR, Boynton MH, Pepper JK, et al Public understanding of cigarette smoke constituents: Three US surveys. Tob Control. 2016;26(5):592–9. 10.1136/tobaccocontrol-2015-052897 27924009 PMC5495614

[17] Hall MG, Ribisl KM, Brewer NT. Smokers’ and nonsmokers’ beliefs about harmful tobacco constituents: Implications for FDA communication efforts. Nicotine Tob Res. 2014;16(3):343–50. 10.1093/ntr/ntt158 24151139 PMC3920339

[18] Baig SA, Byron MJ, Lazard AJ, Brewer NT. “Organic,” “natural,” and “additive-free” cigarettes: Comparing the effects of advertising claims and disclaimers on perceptions of harm. Nicotine Tob Res. 2018;1: 7 Available from: https://academic.oup.com/ntr/advance-article-abstract/doi/10.1093/ntr/nty036/4909729?redirectedFrom=PDF 10.1093/ntr/nty036 PMC6588395 29529277

[19] Moran MB, Pierce JP, Weiger C, Cunningham MC, Sargent JD. Use of imagery and text that could convey reduced harm in american spirit advertisements. Tob Control. 2016;26(e1):e68–70. 10.1136/tobaccocontrol-2016-053251 27609781 PMC5342944

[20] O’Connor RJ, Lewis MJ, Adkison SE, Bansal-Travers M, Cummings KM. Perceptions of “natural” and “additive-free” cigarettes and intentions to purchase. Health Educ Behav. 2016;44(2):222–6. 10.1177/1090198116653935 27281493 PMC6390277

[21] Epperson AE, Henriksen L, Prochaska JJ. Natural American spirit brand marketing casts health halo around smoking. Am J Public Health. 2017;107(5):668–70. 10.2105/AJPH.2017.303719 28398789 PMC5388969

[22] Pepper JK, Byron MJ, Ribisl KM, Brewer NT. How hearing about harmful chemicals affects smokers’ interest in dual use of cigarettes and e-cigarettes. Prev Med. 2017;96:144–8. 10.1016/j.ypmed.2016.12.025 28024860 PMC5329070

